# Effects of Ensiling *Oxytropis glabra* with Whole-Plant Corn at Different Proportions on Fermentation Quality, Alkaloid Swainsonine Content, and Lactic Acid Bacteria Populations

**DOI:** 10.3390/ani10101733

**Published:** 2020-09-24

**Authors:** Ya Tao, Dongze Niu, Feng Li, Sasa Zuo, Qizhong Sun, Chuncheng Xu

**Affiliations:** 1 College of Engineering, China Agricultural University, Beijing 100083, China; taoya@caas.cn (Y.T.); zss@cau.edu.cn (S.Z.); 2 Grassland Research Institute, Chinese Academy of Agricultural Sciences, Hohhot 010010, China; lifeng03@caas.cn (F.L.); sunqz@126.com (Q.S.); 3 Institute of Urban and Rural Mining, Changzhou University, Changzhou 213164, China; ndz@cau.edu.cn

**Keywords:** innovative forage, microorganism, silage quality, swainsonine detoxification, *Zea mays* L.

## Abstract

**Simple Summary:**

In Inner Mongolia, developing innovative forages is an indispensable way to ease the shortage of animal feed. *Oxytropis glabra* (OG) has become a dominant population, with a high nature yield in the degraded grassland of Inner Mongolia. As a typical legume, it is rich in amino acids and trace elements, so using OG to feed livestock is a valuable strategy. However, it contains swainsonine (SW), which hinders the utilization of OG. This study was conducted to evaluate silage fermentation and SW removal from OG ensiled with whole-plant corn at different proportions, and the result showed ensiling a mixture of OG and corn could be a valuable approach for producing livestock feed, as it had a positive effect on fermentation quality and SW detoxification; the optimal ratio was 1:1. In the silages, *Lactobacillus plantarum* was the most common microorganism present in all mixture silages, and *Lactobacillus amylovorus* and *Lactobacillus* *brevis* were prevalent at lower ratios of corn to OG. All representative strains were able to remove SW from OG fermentation, and the strains of *L. amylovorus* and *L. plantarum* had a higher SW removal rate. These mixtures of silages could make OG useable as a feed source in ruminant production.

**Abstract:**

*Oxytropis glabra* (OG) is a leguminous forage that is potentially valuable for solving the shortage of feed for livestock production, while, in large quantities, it may be toxic because of its swainsonine (SW) content. In this study, OG was ensiled with whole-plant corn (*Zea mays* L.) at 10:0, 9:1, 8:2, 7:3, 6:4, 5:5, 4:6, and 0:10 ratios on a fresh matter basis, and, after 60 d of ensiling, the chemical composition, fermentation characteristic, SW removal rate, lactic acid bacteria (LAB) populations, and their capabilities for SW removal were analyzed. As the proportion of corn in the silage increased, the pH, as well as the propionic acid, ammonia-N, dry matter, crude protein, and SW contents, decreased linearly, while the lactic acid, neutral detergent fiber, and residual water-soluble carbohydrate contents increased linearly. *Lactobacillus plantarum* was the most common microorganism present in all mixture silages. *Lactobacillus amylovorus* and *Lactobacillus* *brevis* were prevalent at lower ratios of corn to OG. Meanwhile, the LAB strains belong to *L. amylovorus* and *L. plantarum* had a higher SW removal rate. Our results suggested that ensiling OG with whole-plant corn improves fermentation and decreases SW content, and that 5:5 is the optimal ratio, so this type of mixed silage could make OG useable for ruminant production.

## 1. Introduction

Animal husbandry has always been a leading industry in western Inner Mongolia, and the number of livestock has reached around 126 million in recent years. Traditional feed resources for local ruminants are dominated by grazing in native grassland, which has been unable to meet the requirement of animal feed, especially in central and western Inner Mongolia, the arid conditions lead to limit the growth of forage and reduce the supplying of feed for livestock. Although forage crops, such as corn and alfalfa, had been introduced into the animal production system, it is also a particular need to develop some innovative forages for ruminant production. *Oxytropis glabra* (OG) is a perennial locoweed belonging to the *Oxytropis* genus, and grows widely in western China. Its hardy root system and high resistance to stress have enabled dominant OG populations with high natural yields to develop in the degraded grasslands of Inner Mongolia. OG is a typical legume, rich in amino acids and trace elements [1,2], therefore, the utilization of OG as ruminant feed is a valuable strategy for alleviating forage deficits in winter and spring and effectively increasing the income of local herders [3]. However, OG often contains the toxic alkaloid swainsonine (SW), which is an α-mannosidase and mannosidase II inhibitor. Excessive intake can alter glycoprotein processing and cause lysosomal storage disease [4,5]. Thus, the content of SW hinders OG utilization and can cause serious economic loss [2]. Detoxification could therefore enhance the rate of OG utilization. Some toxic plants have been exploited as feed sources after detoxification, as they still retain their nutrients and economic value [6,7]. However, detoxifying OG for use as a feed source has received relatively little attention.

Biodegradation, which detoxifies environmental toxins by introducing microorganisms, is an efficient way of reducing levels of the target chemicals. Lactic acid bacteria (LAB) can remove aflatoxin, chlorpyrifos, N-nitrosodimethylamine, deoxynivalenol, and other toxins from food and silage by adsorption or metabolism [8,9,10,11]. Some microorganisms, such as *Acinetobacter calcoaceticus* and *Stenotrophomonas maltophilia*, can degrade SW [12,13] Hence, LAB, a key player in the fermentation process, and some of the other microbes typically found in silage may be able to detoxify SW [14,15].

Ensiling is the most used technique for conserving ruminant feed currently, because it minimizes the loss of nutrients from harvest through storage, allows for easier feeding, and often allows for more efficient and timely feed mixing and handling on the farm than hay [16]. The forage preservation method also changes several compounds and sensory characteristics of the animal origin product. Serrapica et al. [17] found a silage based diet induced higher perceived intensity of several attributes. Hence, silage has become the predominant dietary ingredients used in livestock production. Generally, it is difficult to successfully ensile legume forages because of their low fermentable carbohydrate content and high buffering capacity (BC) [18]. Ensiling forage mixtures has recently emerged as a feasible strategy for improving the fermentation of legumes [19,20]. Corn (*Zea mays* L.) is easier to ferment, because of its high water-soluble carbohydrate (WSC) content, and adding it to legume silage improves fermentation quality. While altered fermentation profiles have been demonstrated when corn is ensiled with different legume species [21,22], and little is known about the feasibility of ensiling OG with whole-plant corn.

The aim of this study was to evaluate silage fermentation and SW removal from OG ensiled with whole-plant corn at different proportions, and to identify the optimal ratio of OG to corn. Moreover, the effects of LAB on fermentation quality in different mixture silages and their capability of SW removal were also investigated, to support later studies on the removal mechanism of SW in the ensiling process.

## 2. Materials and Methods

### 2.1. Silage Preparation

*Oxytropis glabra* was collected from natural grasslands (38°59′ N, 108°84′ E) in Wuchen Banner, Ordos, China (locates in the main distribution area of *Oxytropis glabra*, and with *Stipa bungeana* Trin., *Artemisia ordosica* Krasch. and some shrubs as the common plants) at the blooming stage (50% flowering rate) on 12 August 2016, when corn was at the early milky stage in this region. In order to remain in line with the actual situation of the local production, whole corn plants were harvested at the early milky stage from nearby farmland on the same day, rather than at the optimal harvest time (>34% DM). About 30 kg of OG and whole-plant corn (fresh biomass) were harvested at multi-point by scythe, leaving a 5 cm stubble above the ground level, and chopped into 2 cm lengths, then combined at ratios of 10:0, 9:1, 8:2, 7:3, 6:4, 5:5, 4:6, and 0:10 of OG to corn, based on fresh weight. For each combination, 5 kg mixture were prepared. Before ensiling, 500 g of initial fresh material in each mixture were sampled for microbial and chemical analysis. Approximately 500 g samples of remaining mixtures were weighed into individual silo bags (250 × 360 mm, Shijiazhuang Xilong Packing Co., Ltd., Shijiazhuang, China) in triplicate and sealed with a vacuum sealer (DZ-260, Beijing Jod Packing Machinery Co., Ltd., Beijing, China). The silo bags were then stored at room temperature (20–25 °C) for 60 d.

### 2.2. Microbial and Chemical Analysis

The microbial populations of the fresh materials and silages were measured by the plate count method. Samples were blended and serially diluted in sterilized water. The numbers of LAB, coliform bacteria, aerobic bacteria, mold, and yeast colonies were counted according to Yang et al. [23]. For isolation of LAB, 5–10 colonies with different appearance were picked randomly on MRS agar medium from each sample of fresh materials and their corresponding silages. They were preliminarily determined to be LAB, based on cell morphology, Gram stain appearance, catalase test, and lactic acid productivity, then purified by streaking twice to de Man, Rogosa and Sharpe (MRS) agar (Difco, Detroit, MI, USA). The purified LAB cultures were suspended in a solution of nutrient broth (Difco) and dimethyl sulfoxide at a ratio of 9:1, and stored at −80 °C for 16S rRNA gene sequencing.

Twenty grams of each silage sample were homogenized with 180 mL of distilled water, then filtered through four layers of cheesecloth. The pH of the filtrates was measured using a glass electrode pH meter (UB-7, Denver Instruments, Denver, CO, USA), and the ammonia-N concentrations were analyzed according to Broderick and Kang [24]. The organic acid contents were determined by high-performance liquid chromatography (HPLC; LC-10A, Shimadzu, Kyoto, Japan; column: Shodex RS Pak KC-811, Showa Denko K.K., Tokyo, Japan; detector: DAD, 210 nm, SPD-20A; eluent: 3 mmol/L HClO_4_, 1.0 mL/min; temperature: 50 °C). Dry matter (DM) content was determined by oven drying at 65 °C for 48 h, and the dried samples then ground with a cutting mill (SM100; Retsch Technology, Haan, Germany) to 1 mm for further chemical analysis. Crude protein (CP) content was analyzed according to AOAC methods [25]. The neutral detergent fiber (NDF) and acid detergent fiber (ADF) contents were measured according to Van Soest et al. [26]. WSC content was determined using the anthrone method [27].

Swainsonine was extracted according to Hao et al. [28]. Five grams of dry and ground plant material was extracted with petroleum ether (100 mL) for 1 h at 40 °C and 40 kHz, using ultrasound extraction. The solvent was then removed by rotary evaporation to obtain a syrup, which was then saturated with 30 mL of methanol and subjected to ultrasound extraction for 3 h. An aliquot of the methanolic extract was dried by rotary evaporation. The residue was dissolved in 1 mol/L HCl and then centrifuged to remove impurities. The supernatant was extracted thrice more with chloroform, and the pH of the aqueous layer was adjusted to 10.0 using 1 mol/L NaOH, then the aqueous layer was extracted thrice more with n-butanol. The n-butanol was removed by rotary evaporation, and the gummy residue was dissolved in 2 mL of pyridine. Fifty microliters BSTFA + TMCS (BSTFA:TMCS = 99:1) and 0.2 mg internal standard (me-gal) were added into 50 μL pyridine-dissolved sample, and derivatived at room temperature for 1 h. One microliter of the aliquots was analyzed by a gas chromatograph (GC, Varian 3800, Varian Inc., Walnut Creek, USA) equipped with a flame ionization detector (FID) and an AT.SE-54 column. The column temperature was kept at 210 °C, the injector port temperature was at 260 °C and the detector block at 280 °C. Purified dry nitrogen was used as carrier gas at a flow ratio of 2 mL/min; the split rate was 30:1, as previously described [13].

### 2.3. Species Identification by 16S rRNA Sequencing

Five milliliters of LAB, isolated from OG and whole-plant corn mixtures and their silages, cultured overnight at 37 °C in MRS medium was used for DNA extraction and purification. Genomic DNA was extracted using a TIANamp Bacterial DNA kit (Tiangen Biotech, Beijing, China), according to the manufacturer’s instructions. The 16S rRNA gene sequence coding region was amplified with the primers 27f (5′-AGA CTT TGA TCC TGG CTC AG-3′) and 1492r (5′-TAC GGC TAC CTT GTT ACG ACT-3′), using 2xTaq PCR MasterMix (Tiangen) and a polymerase chain reaction (PCR) thermal cycler (Veriti 96-Well Thermal Cycler, Thermo Fisher Scientific, Singapore) [29]. The PCR products were sequenced by Personalbio Co., Ltd. (Shanghai, China) and compared with sequences from the type strains available in GenBank using the Basic Local Alignment Search Tool (BLAST). Nucleotide substitution rates (*K*nuc values) were calculated [30], and phylogenetic trees were constructed using the neighbor-joining method [31] based on the 16S rRNA gene sequences from the isolates and correlative type strains. *Bacillus subtilis* DSM 10^T^ was used as an outgroup organism. The topologies of the trees were evaluated by bootstrap analysis of the sequence data using Molecular Evolutionary Genetic Analysis (MEGA) 4 software (The Biodesign Institute, Tempe, AZ, USA), based on random resampling with 1000 repetitions [32].

### 2.4. Determination of the SW Removal Rate in OG Broth by LAB

Fresh OG material was mixed with deionized water at 1:1 ratio, then squeezed. The OG broth was prepared after filtering and sterilizing. One to four representative LAB strains of each species, isolated from OG and whole-plant corn mixtures and their silages, were picked out randomly, then cultivated in MRS broth overnight, and adjusted the cell concentration to 1.0 × 10^9^ cfu/mL. Three milliliters bacterial suspensions was centrifuged to discard the supernatant, and the strain was mixed into 3 mL OG broth, a control was added with no LAB strains, then fermented at 37 °C for 48 h. Each strain was analyzed in triplicate. The content of SW in OG broth was determined by GC as above, and the SW remove rate was calculated as follows:

$$\mathrm{SW}\;\mathrm{romoval}\;\mathrm{rate}=\frac{\mathrm{CSW}\;-\;\mathrm{OSW}}{\mathrm{CSW}}\times 100$$

where CSW is the SW content in the control broth; OSW is the SW content in the fermented OG broth by LAB according to the methods of Qi et al. [33].

### 2.5. Statistical Analysis

Microbial counts were converted to log_10_, and the results were expressed on a fresh matter (FM) basis. Treatment effects were evaluated by one-way analysis of variance (ANOVA) using SPSS software (SPSS 24.0, SPSS, Inc., Chicago, IL, USA). The means were compared for significance by Tukey’s honest significance test. Polynomial contrasts were used to test linear, quadratic, and cubic responses to whole-plant corn mixing ratios. The results were considered statistically significant at *p* < 0.05.

## 3. Results

### 3.1. Microbial Populations of OG and Whole-Plant Corn Mixtures Prior to and after 60 d of Ensiling

There was a linear increase (*p* < 0.05) in the LAB and yeast populations, but a linear decrease (*p* < 0.05) in the aerobic bacteria population as the ratio of corn to OG increased in fresh materials prior to ensiling (Table 1). A quadratic response (*p* < 0.05) in the LAB, aerobic bacteria, and yeast populations, and a cubic response (*p* < 0.05) in the yeast population were observed. When the ratio of OG to corn was 8:2, and at ratios with greater corn contents, the LAB populations exceeded 5.00 log_10_ cfu/g FM. There were no significant differences in the coliform bacteria and mold populations among the raw material mixtures (*p* > 0.05).

After 60 d of ensiling, the LAB populations reached 6.16–6.97 log_10_ cfu/g FM, the coliform bacteria and mold populations declined below the detection limits, and there was no notable difference in the LAB, aerobic bacteria, and yeast populations among all mixture silages (*p* > 0.05).

### 3.2. Chemical Compositions of OG and Whole-Plant Corn Mixtures Prior to and after 60 d of Ensiling

As the corn ratio of the forage mixture increased, the DM and CP contents decreased, but the NDF and WSC contents increased linearly prior to ensiling (*p* < 0.05). Moreover, the ADF content exhibited a quadratic response (*p* < 0.05), and DM, CP, NDF, and WSC exhibited quadratic and cubic responses (*p* < 0.05) to increasing the corn ratio in the mixtures (Table 2).

After ensiling, increasing the proportion of corn in the mixture silages resulted in a linear decrease (*p* < 0.05) in the DM and CP contents, but a linear (*p* < 0.05) increase in the NDF and WSC contents. In addition, the WSC content exhibited a cubic response (*p* < 0.05), and the CP and NDF contents exhibited quadratic and cubic responses (*p* < 0.05). There was no significant difference in ADF content among the mixture silages (*p* > 0.05).

### 3.3. Fermentation Characteristics of OG and Whole-Plant Corn Mixture Silages

Compared with OG silage (10:0), the propionic acid and ammonia-N contents were lower (*p* < 0.05) in silages containing whole-plant corn. The highest pH was detected in OG silage (10:0), and this pH value was significantly higher (*p* < 0.05) than that observed in silages with 5:5 and 6:4 ratios of OG to corn. As the amount of corn in the mixture silages increased, the pH, propionic acid and ammonia-N contents decreased linearly (*p* < 0.05), and the lactic acid content increased linearly (*p* < 0.05); only minor differences in the lactic acid and acetic acid contents were observed among all of the mixture silages (Table 3). In addition, increasing the proportion of corn in the mixtures led to a quadratic response (*p* < 0.05) in pH and propionic acid content, and a cubic response (*p* < 0.05) in the propionic acid and ammonia-N contents of the mixture silages. Butyric acid was not detected in any of the mixtures after ensiling.

### 3.4. Isolation, 16S rRNA Gene Sequencing, and Phylogenetic Analysis of LAB

**Figure 1:**
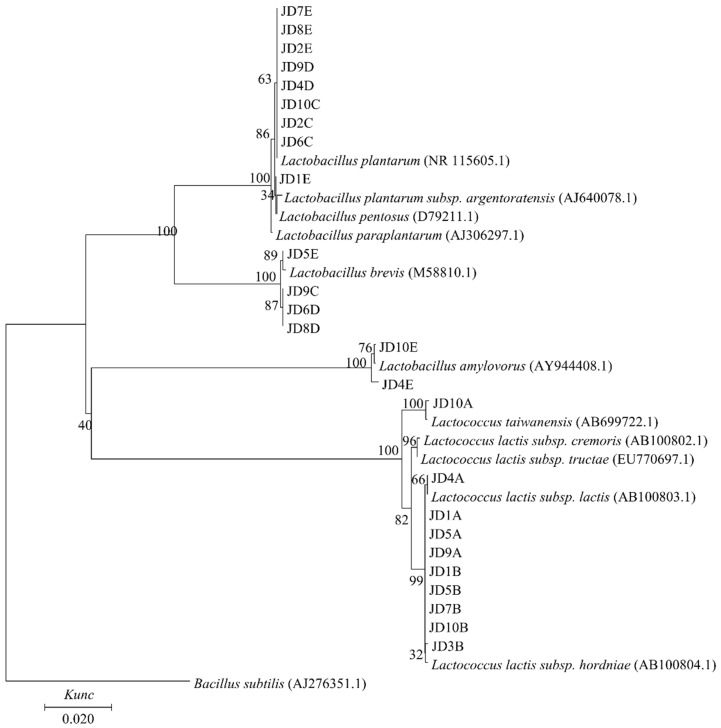
Phylogenetic tree showing the relative position of strains isolated from raw *Oxytropis glabra*-whole-plant corn mixtures and their silages. The tree was constructed using the neighbor-joining method with 16S rRNA gene sequences. Bootstrap values for a total of 1000 replicates are shown at the nodes of tree. *Bacillus subtilis* was used as an outgroup. The bar indicates 1% sequence divergence; *Kunc* nucleotide substitution rates.

Phylogenetic analysis was conducted based on the 16S rRNA gene sequence of the LAB strains isolated from the raw OG and whole-plant corn mixtures and their silages. All strains could be divided into seven different groups. The representative strains of different groups in each treatments were listen in the Table 4, and the phylogenetic tree of their 16S rRNA gene sequences are shown in Figure 1. All LAB strains could be grouped into two different genera. Strains JD7E, JD8E, JD2E, JD9D, JD4D, JD10C, JD2C, and JD6C were phylogenetically closest to *Lactobacillus plantarum*, with 99.79–100.00% 16S rRNA gene sequence similarity. Strain JD1E clustered with *L. plantarum, Lactobacillus pentosus*, and *Lactobacillus plantarum* subsp. *argentoratensis*, with 16S rRNA gene sequence similarities of 100.00%, 100.00%, and 99.38%, respectively, so this strain belonged to *L. plantarum* group. Strains JD5E, JD9C, JD6D, and JD8D were most closely related to *Lactobacillus brevis*, as supported by a 100.00% bootstrap value. Strains JD10E and JD4E were grouped into a 100.00% bootstrap cluster with *Lactobacillus amylovorus*. Strains JD10A and JD4A grouped with *Lactococcus taiwanensis* and *Lactococcus lactis subsp. lactis*, respectively, with 99.86% and 99.93% similarity for the 16S rRNA gene sequence, respectively, and 100.00% bootstrap values. Strains JD1A, JD5A, JD9A, JD1B, JD3B, JD5B, JD7B, and JD10B were closely related to *Lactococcus lactis subsp. hordniae*, and their 16S rRNA gene sequences had high similarity to their type strains (>99.79%).

### 3.5. SW Content and Removal Rate of OG and Whole-Plant Corn Mixture Silages

**Figure 2:**
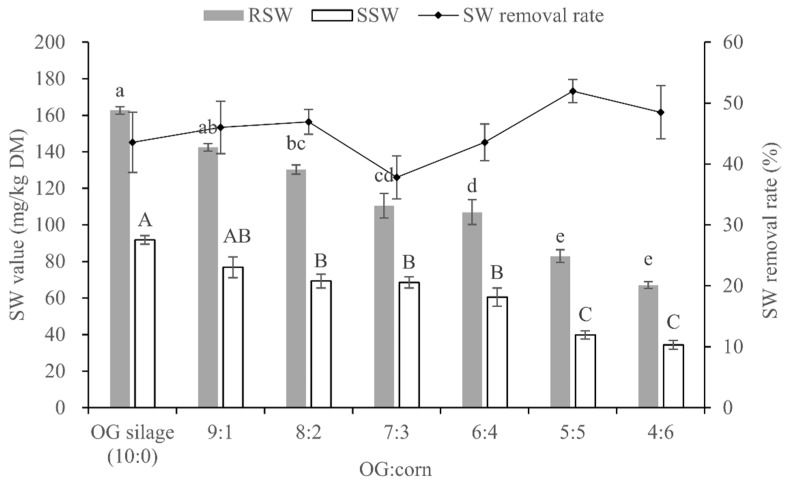
Swainsonine (SW) content prior to and after ensiling of *Oxytropis glabra* (OG) mixed with whole-plant corn at different ratios, and the rate of SW removal from the silages. Means without a common superscript letter differ (*p* < 0.05); error bars denote the standard error of three replicates. RSW, SW content of raw materials; SSW, SW content of silages. Polynomial contrasts: RSW, *p* < 0.001 (linear), *p* = 0.893 (quadratic), *p* = 0.251 (cubic); SSW, *p* < 0.001 (linear), *p* = 0.361 (quadratic), *p* = 0.222 (cubic).

The SW contents of the raw mixtures ranged from 66.98 to 162.64 mg/kg on a DM basis, and as the amount of corn in the mixtures increased, the SW content decreased linearly (*p* < 0.05, Figure 2). After 60 d of ensiling, the SW contents decreased and ranged from 34.41 to 91.75 mg/kg on a DM basis, and as the amount of corn in the mixture silages increased, the SW content decreased linearly (*p* < 0.05) too. There were no significant differences in SW removal rate among the different mixture silages (*p* > 0.05).

### 3.6. The SW Removal Rate in OG Broth by LAB Strains

The SW removal rates of all representative LAB strains were higher than 85% after fermentation for 48 h in OG broth, and the strains JD10E, JD2E, JD4D, JD6C, and JD10C have a greater (*p* < 0.05) SW removal rate, up to 100% (Table 5).

## 4. Discussion

Microorganisms naturally present on forage are responsible for silage fermentation, but can be affected by many factors and vary widely [34]. Only 4.00 log_10_ cfu/g FM of LAB was present in the raw OG (Table 1), which was inadequate for natural fermentation during ensiling [35]. In contrast, the fresh whole-plant corn contained 5.64 log_10_ cfu/g FM of LAB and relatively lower number of aerobic bacteria compared with the OG, which was sufficient for improving the silage quality. In addition to LAB, the properties of the raw material also determine the rate of pH decline at early stages of ensiling. For optimal fermentation, silage should contain 60–70 g/kg DM of WSC as a substrate for LAB [36], and when the ratio of OG to corn was 7:3 or the corn content was even higher, the raw material mixtures were suitable for producing high-quality silage. Hence, a linear increase in WSC and LAB in the raw mixtures due to the addition of corn led to an improvement in the quality of the mixture silages, with a linear decrease in pH, propionic acid content, and ammonia-N content, and a linear increase in lactic acid content. After 60 d of ensiling fermentation, the LAB populations in all treatment silages reached about 6.00 log_10_ cfu/g FM (Table 1). In agreement with our findings, Chen et al. [37] showed when ensiling different ratios of whole crop oat to Lucerne, all silages were well preserved with low pH and LAB were stable around 6.00 log_10_ cfu/g FM. Coliform bacteria and mold were not detected, and only small differences in the relative numbers of other microflora were observed in all of the mixture silages, indicating full fermentation [38]; this can be attributed to the minimal differences in lactic acid and acetic acid contents in all of the mixture silages. The high protein concentration in legumes contributes to a high BC, and results in high resistance to changes in pH. Consistent with this, although no significant difference in lactic acid and acetic acid levels were observed among the mixture silages, the highest pH was detected in the OG-only silage. The pH values of 4.24 or less observed in mixture silages containing whole-plant corn almost met the requirements for good quality silage [14], especially when OG ensiled at 5:5 and 4:6 ratio with whole-plant corn, both of these conditions exhibited the lowest (*p* < 0.05) pH values among the mixture silages, but the pH in the OG-only silage did not decrease to this level, which resulted in greater ammonia-N production in the OG silage than in silages containing whole-plant corn.

Among epiphytic LAB, lactic acid-producing cocci begin the silage fermentation process, creating an aerobic environment that is suitable for the proliferation of lactobacilli, and cocci are only prevalent in the early stages of ensiling [39]. *Lactococcus* was the major component of the LAB presented on OG and whole-plant corn. *Lac. lactis* subsp. *lactis* is commonly detected on plant material [40], while, in the present study, *Lac. lactis* subsp. *hordniae* was the predominant species found on OG, whole-plant corn, and in the OG-whole-plant corn mixtures, and *Lac. lactis* subsp. *lactis* was only found on whole-plant corn. To our knowledge, this is the first report of natural *Lac. lactis* subsp. *hordniae* isolated from forage. In most respects, the characteristics of *Lac. lactis* subsp. *hordniae* correspond to those of *L. lactis subsp. lactis* [41].

After fermentation, *Lactobacillus* dominated the silages. This genus is always present in silage, and improves fermentation quality. *L. plantarum* was one of the dominant LAB present in all of the silages, and, given its promotion of lactic acid fermentation over long periods of time, it was likely responsible for the minimal variation in the lactic acid content among the silages. Although individual bacterial species were detected in the raw materials, adding whole-plant corn led to greater diversity in the LAB species present throughout the ensiling processes. In addition to *L. plantarum*, *L. amylovorus* dominated OG silage and the silage containing OG and corn at a ratio of 9:1. The dominance of *L. amylovorus* was most likely due to the low WSC content of these raw materials (<6.0% DM), as this species can utilize a wider range of substrates, such as cellobiose and starch [42], to improve fermentation. This could account for the minor differences in pH between the OG and whole-plant corn silages, although whole-plant corn contains more WSC than OG does. Moreover, *L. brevis* was detected in silage with a 9:1 ratio of OG to corn, due to having a sufficient amount of substrate, and gradually overtook *L. amylovorus* to occupy the main ecological niche as the relative amount of corn in the silage increased. In well-fermented silages, *L. plantarum* predominates the early stages (4–7 d) of ensiling, then more species, such as *L. brevis*, *L. homohiochii*, and *L. gasseri*, appear and become prevalent, depending on the crop [18]. When the corn proportion exceeded half of the mixture, *L. plantarum* was the only dominant species in the silages and was accompanied by proper fermentation, which is consistent with Lin et al. [18], who showed that homofermentative LAB species accumulate during ensiling and are ultimately responsible for corn fermentation. The greater the size of the homofermentative LAB population involved in ensiling, the greater the chance of producing well-preserved silage. Moreover, at an OG to corn ratio of 8:2, *L. amylovorus* and *L. brevis* could not be detected, and were taken over by the stains JD1E in the silage. It belongs to *L. plantarum* group, but could not be identified to species level for high identity value of 16S rRNA gene sequence shared by *L. plantarum, L. pentosus and L. plantarum subsp. argentoratensis*, and more complicated alternative methods, such as *recA* gene sequence analysis are necessary in further study [43]. Perhaps because epiphytic LABs are sensitive to the ensiling environment, some conditions resulted in different LAB species or strains being dominant in this mixture silage [18]. In summary, ensiling OG mixed with whole-plant corn at different ratios affected the dominant LAB present within each mixture silage. Presumably because of differences in substrate, moisture, and other conditions, *L. amylovorus* and *L*. *brevis* were prevalent in mixture silages with different ratios of corn to OG, in addition to *L. plantarum*, which was present in all of the silages tested. When the corn content reached a certain threshold, the silages were dominated by *L. plantarum* alone.

The SW content in the OG and whole-plant corn mixture silages declined by 37.81–51.96% relative to the fresh materials, and when the proportion of corn in the mixtures were higher than 5:5, the SW contents in silages were lower than 40.00 mg/kg of DM, which were less than a quarter of that in fresh OG. Ensiling has also been shown to decrease the contents of other toxins. Huisden et al. [6] found that the L-3,4-dihydroxyphenylalanine content decreased by 54% during *Mucuna pruriens* ensiling. Additionally, Wang et al. [44] showed that the DM, CP and minerals digestibility of corn stalk silage to karakul rams could be improved when it was displaced by 14% of fresh OG for three weeks, without health harmful. Hence, ensiling can be used to improve the feeding scale of OG in ruminant diets as forage. Microorganisms are able to detoxify toxins in contaminated environments to protect themselves. Wang et al. [9] found that, during ensiling, LABs and other microbes play a major role in chlorpyrifos removal, and that the ensiling inoculated with *L. casei* led to more efficient adsorption or metabolism of chlorpyrifos. Microbial consortia could be primarily responsible for SW removal from OG and whole-plant corn mixture silages. The small differences in microbial populations among the mixture silages (Table 1) suggested that the differences in SW removal contents among the silages probably because of the presence of different microbial species, rather than different microbial numbers. The 1,2-di-hydroxyl structure of SW is relatively unstable and may be oxidized by alcohol dehydrogenases [45]. Nicotinamide adenine dinucleotide phosphate (NADP)-dependent alcohol dehydrogenase has a strong ability to degrade SW [46], and this enzyme is produced by some LAB species, such as *Leuconostoc cremoris* [47]. In this study, the SW removal capability of LAB belonging to different species in fresh materials and silages has been explored preliminarily. All representative strains of each species were able to remove SW from OG broth after fermentation for 48 h, especially the strains belonging to *L. amylovorus* and *L. plantarum*, which performed better. Thus, the LAB strains present in these silages may also play a key roles in SW removal, and the detoxification mechanisms of specific strains, adsorption or metabolism, should be investigated further. However, care should be taken to ensure that total SW intake does not exceed safe levels, and further research is needed to determine the safe consumption of OG silage in an optimal mixture ratio.

## 5. Conclusions

In Inner Mongolia, developing innovative forages is an indispensable way to solve the shortage of feed for livestock production. Ensiling the mixtures of OG and whole-plant corn improved the fermentation quality and decreased the SW content, and the silage with a ratio of 5:5 has the potential to be used for ruminant. *L. plantarum* was the most common microorganism present in all mixture silages, and it especially dominated in the silages where the corn proportion accounted for more than half of the mixture, and *L. amylovorus* and *L*. *brevis* were prevalent at lower ratios of corn to OG. All representative strains were able to remove SW from OG fermentation, and the strains of *L. amylovorus* and *L. plantarum* had a higher SW removal rate. These mixture silages could make OG useable as a feed source in ruminant production.

## Figures and Tables

**Table 1:** Microbial populations (log_10_ cfu/g FM) of *Oxytropis glabra* (OG) mixed with whole-plant corn at different ratios prior to and after 60 d of ensiling.

OG:Corn (Percentage of FM)	Prior to Ensiling					60 d of Ensiling				
	LAB	CB	AB	Yeast	Mold	LAB	CB	AB	Yeast	Mold
OG silage (10:0)	4.00 ^b^	5.16	6.24 ^a^	4.15 ^b^	2.40	6.97	ND	6.30	6.40	ND
9:1	4.64 ^ab^	5.27	6.47 ^a^	5.38 ^a^	2.70	6.58	ND	6.02	5.06	ND
8:2	5.45 ^a^	5.17	6.51 ^a^	5.68 ^a^	2.55	6.28	ND	5.80	5.56	ND
7:3	5.67 ^a^	5.25	6.65 ^a^	5.87 ^a^	2.40	6.88	ND	6.56	5.21	ND
6:4	5.71 ^a^	4.79	6.46 ^a^	5.78 ^a^	2.55	6.16	ND	6.04	4.80	ND
5:5	5.56 ^a^	4.93	6.24 ^a^	5.76 ^a^	2.40	6.31	ND	5.98	4.86	ND
4:6	5.44 ^a^	4.81	5.15 ^b^	5.69 ^a^	2.55	6.23	ND	5.43	5.55	ND
Corn silage (0:10)	5.64 ^a^	4.70	5.33 ^b^	6.01 ^a^	2.85	6.24	ND	5.41	5.80	ND
SEM	0.162	0.104	0.143	0.152	0.048	0.101	-	0.125	0.175	-
Linear	0.001	0.169	<0.001	0.001	0.165	0.048	-	0.061	0.493	-
Quadratic	0.005	0.770	0.001	0.005	0.119	0.521	-	0.299	0.029	-
Cubic	0.145	0.708	0.325	0.010	0.031	0.749	-	0.604	0.945	-

^a–b^ Means within columns with different superscript letters differ (*p* < 0.05). FM, fresh matter; LAB, lactic acid bacteria; CB, coliform bacteria; AB, aerobic bacteria; ND, not detected; SEM, standard error of mean.

**Table 2:** Chemical compositions (% DM) of *Oxytropis glabra* (OG) mixed with whole-plant corn at different ratios prior to and after 60 d of ensiling.

OG:Corn (Percentage of FM)	Prior to Ensiling					60 d of Ensiling				
	DM	CP	NDF	ADF	WSC	DM	CP	NDF	ADF	WSC
OG silage (10:0)	27.11 ^a^	19.10 ^a^	43.04 ^e^	37.36 ^a^	5.26 ^c^	26.31 ^a^	18.17 ^a^	42.64 ^e^	36.36	1.35 ^c^
9:1	27.15 ^a^	18.55 ^ab^	45.66 ^d^	35.91 ^ab^	5.16 ^c^	26.42 ^a^	18.15 ^a^	43.69 ^e^	35.18	2.21 ^b^
8:2	26.22 ^b^	18.19 ^b^	46.36 ^d^	35.14 ^b^	6.11 ^bc^	25.07 ^b^	16.30 ^b^	45.63 ^de^	35.78	2.23 ^b^
7:3	24. 70 ^c^	17.18 ^c^	47.13 ^cd^	36.21 ^ab^	7.27 ^b^	24.12 ^bc^	15.59 ^bc^	46.85 ^cd^	35.90	2.20 ^b^
6:4	23. 97 ^d^	15.27 ^d^	48.99 ^bc^	35.68 ^ab^	7.24 ^b^	23.24 ^cd^	14.45 ^cd^	49.04 ^bc^	35.12	2.60 ^b^
5:5	23.67 ^e^	13.79 ^e^	49.15 ^bc^	36.28 ^ab^	6.99 ^b^	22.40 ^de^	13.51 ^de^	50.22 ^b^	36.51	2.50 ^b^
4:6	23.33 ^f^	13.51 ^e^	50.31 ^b^	35.48 ^ab^	7.01 ^b^	22.30 ^de^	13.01 ^e^	50.75 ^b^	34.81	2.61 ^b^
Corn silage (0:10)	22.60 ^g^	8.23 ^f^	57.73 ^a^	36.27 ^ab^	10.31 ^a^	21.31 ^e^	8.65 ^f^	58.33 ^a^	35.43	3.59 ^a^
SEM	0.427	0.877	1.060	0.186	0.394	0.379	0.615	0.995	0.175	0.143
Linear	<0.001	< 0.001	<0.001	0.167	<0.001	<0.001	<0.001	<0.001	0.224	<0.001
Quadratic	<0.001	< 0.001	<0.001	0.020	0.008	0.361	<0.001	0.001	0.924	0.469
Cubic	<0.001	< 0.001	<0.001	0.071	<0.001	0.089	0.002	0.003	0.303	<0.001

^a–g^ Means within columns with different superscript letters differ (*p* < 0.05). FM, fresh matter; DM, dry matter; CP, crude protein; NDF, neutral detergent fiber; ADF, acid detergent fiber; WSC, water-soluble carbohydrate; SEM, standard error of mean.

**Table 3:** Fermentation quality of *Oxytropis glabra* (OG) mixed with whole-plant corn after 60 d of ensiling

OG: Corn (Percentage of FM)	pH	Lactic Acid	Acetic Acid	Propionic Acid	Butyric Acid	Ammonia-N (% TN)
		(% DM)				
OG silage (10:0)	4.46 ^a^	2.20	1.71	0.15 ^a^	ND	4.86 ^a^
9:1	4.22 ^ab^	3.15	1.39	0.05 ^b^	ND	4.31 ^b^
8:2	4.24 ^ab^	3.59	1.41	0.02 ^b^	ND	4.20 ^b^
7:3	4.24 ^ab^	4.01	1.26	0.06 ^b^	ND	4.27 ^b^
6:4	4.22 ^ab^	4.46	1.19	0.00 ^b^	ND	4.21 ^b^
5:5	4.21 ^b^	3.79	1.47	0.00 ^b^	ND	4.16 ^b^
4:6	4.21 ^b^	4.56	1.19	0.00 ^b^	ND	4.06 ^bc^
Corn silage (0:10)	4.24 ^ab^	3.88	1.15	0.00 ^b^	ND	3.71 ^c^
SEM	0.023	0.229	0.086	0.011	-	0.072
Linear	0.022	0.013	0.199	<0.001	-	<0.001
Quadratic	0.016	0.057	0.632	0.001	-	0.373
Cubic	0.145	0.821	0.503	0.045	-	<0.001

^a–c^ Means within columns with different superscript letters differ (*p* < 0.05). FM, fresh matter; TN, total N; ND, not detected; SEM, standard error of mean.

**Table 4:** Strains isolated from raw *Oxytropis glabra* (OG)-whole-plant corn mixtures and their silages

Source	OG: Corn (Percentage of FM)	Strains	Group
Fresh material	OG (10:0)	JD10B	*Lac. lactis subsp. hordniae*
	9:1	JD7B	*Lac. lactis subsp. hordniae*
	8:2	JD5B	*Lac. lactis subsp. hordniae*
	7:3	JD3B	*Lac. lactis subsp. hordniae*
	6:4	JD1B	*Lac. lactis subsp. hordniae*
	5:5	JD9A	*Lac. lactis subsp. hordniae*
		JD10A	*Lac. taiwanensis*
	4:6	JD5A	*Lac. lactis subsp. hordniae*
	Corn (0:10)	JD1A	*Lac. lactis subsp. hordniae*
		JD4A	*Lac. lactis subsp. lactis*
60 d silage	OG silage (10:0)	JD8E	*L. plantarum*
		JD10E	*L. amylovorus*
	9:1	JD4E	*L. amylovorus*
		JD5E	*L. brevis*
		JD7E	*L. plantarum*
	8:2	JD1E	*L. plantarum, L. pentosus*, *L. plantarum* subsp. *argentoratensis*
		JD2E	*L. plantarum*
	7:3	JD8D	*L. brevis*
		JD9D	*L. plantarum*
	6:4	JD4D	*L. plantarum*
		JD6D	*L. brevis*
	5:5	JD9C	*L. brevis*
		JD10C	*L. plantarum*
	4:6	JD6C	*L. plantarum*
	Corn silage (0:10)	JD2C	*L. plantarum*

FM, fresh matter; Lac., Lactococcus; L., Lactobacillus.

**Table 5:** Swainsonine (SW) removal rate (%) in *Oxytropis glabra* (OG) broth by representative LAB strains of different species.

Source	OG: Corn (Percentage of FM)	Strains	Species	SW Removal Rate
Fresh material	5:5	JD9A	*Lac. lactis* subsp. *hordniae*	93.35 ^c^
	4:6	JD5A	*Lac. lactis* subsp. *hordniae*	88.10 ^d^
	Corn (0:10)	JD4A	*Lac. lactis* subsp. *lactis*	85.31 ^e^
60 d silage	OG silage (10:0)	JD10E	*L. amylovorus*	100.00 ^a^
	6:4	JD6D	*L. brevis*	89.12 ^d^
	5:5	JD9C	*L. brevis*	94.96 ^bc^
	8:2	JD1E	*L. plantarum* group	96.72 ^b^
	8:2	JD2E	*L. plantarum*	100.00 ^a^
	6:4	JD4D	*L. plantarum*	100.00 ^a^
	5:5	JD10C	*L. plantarum*	100.00 ^a^
	4:6	JD6C	*L. plantarum*	100.00 ^a^
SEM		-	-	0.591
*p*-value		-	-	<0.001

^a–e^ Means within columns with different superscript letters differ (*p* < 0.05). FM, fresh matter; *Lac.*, *Lactococcus*; *L.*, *Lactobacillus*; SEM, standard error of mean.
